# Risk of Progression and Costs of Care for Patients With Chronic Kidney Disease

**DOI:** 10.1016/j.xkme.2026.101484

**Published:** 2026-08-03

**Authors:** Navdeep Tangri, Rakesh Singh, Keith A. Betts, Yuxian Du, Sophie Gao, Arvind Katta, Youssef M.K. Farag, Samuel T. Fatoba, Hongjiao Liu, Jingyi Chen, Thomas Ferguson, Reid Whitlock, Ryan J. Bamforth, Silvia J. Leon, Ajay K. Singh

**Affiliations:** 1 Department of Community Health Services, Max Rady College of Medicine, University of Manitoba, Winnipeg, MB, Canada; 2 Bayer U.S. LLC, Whippany, NJ; 3 Analysis Group, Inc, Los Angeles, CA; 4 Analysis Group, Inc, Boston, MA; 5 Renal Division, Brigham and Women’s Hospital; Harvard Medical School, Boston, MA

**Keywords:** Chronic kidney disease, CKD progression, economic outcomes, health care costs, health care resource utilization, risk prediction

## Abstract

**Rationale & Objective:**

Chronic kidney disease (CKD) is associated with substantial economic burden. Early identification of high-risk patients with CKD can facilitate timely intervention and reduce CKD-related medical costs. This study assessed the association between CKD progression risk and health care burden.

**Study Design:**

Retrospective observational analysis.

**Setting and Participants:**

1,050,552 adult US patients in Optum’s electronic health records database between January 1, 2007 and September 30, 2022.

**Exposures:**

Risk of CKD progression as determined by a laboratory-based machine learning model.

**Outcomes:**

All-cause health care resource utilization and medical costs.

**Analytical Approach:**

A previously published and validated machine learning model, Klinrisk, was applied to classify patients into 3 groups based on their risk of CKD progression (low, medium, and high). All-cause inpatient admissions, emergency room (ER) visits, and outpatient visits were evaluated in each risk group for the 1 year after CKD diagnosis. Average medical costs (2023 US Dollars) were calculated as the average length of stay for inpatient admissions and average number of ER and outpatient visits multiplied by the corresponding unit costs as estimated from a prior study.

**Results:**

Patients with higher predicted CKD progression risk had higher health care burden. High-risk patients averaged 1.49 inpatient admissions, 0.83 ER visits, and 35.75 outpatient visits per year compared with 0.31 inpatient admissions, 0.63 ER visits, and 24.45 outpatient visits among low-risk patients. The total annual medical costs for low-, medium-, and high-risk patients were $16,018, $22,090, and $50,218, respectively.

**Limitations:**

Our analysis only considered direct medical costs to the payer and omitted indirect costs (eg, transportation, capital, health records). The Klinrisk model only predicts CKD progression, not cardiovascular disease outcomes.

**Conclusions:**

Patients at high risk of CKD progression as predicted by the Klinrisk model had higher health care resource utilization and medical costs and may benefit from medical intervention and care management to reduce economic burden.

## Plain-Language Summary

Klinrisk is a machine learning tool that uses demographics and laboratory results to predict the risk of chronic kidney disease (CKD) progression. The relationship between Klinrisk-predicted CKD progression risk and health care costs across all stages of kidney disease remains unclear. This study utilized electronic health records from more than 1 million patients with CKD in the United States. We found that patients identified as high risk by the model progressed faster than those identified as lower risk. Patients identified as high risk used more health care services and had higher medical costs. Our findings suggest that the Klinrisk model can identify patients at highest risk of CKD progression who may benefit from timely intervention, which may potentially lower health care costs and improve outcomes.

Chronic kidney disease (CKD) is a global health problem associated with significant morbidity and mortality.^1^ Approximately 14% of the US population has CKD, and more than 135,000 patients per year progress to kidney failure, requiring dialysis or a kidney transplant.^1^ Moreover, kidney disease is associated with a substantial burden in terms of health care costs. In the United States, the yearly economic costs of care for Medicare beneficiaries with CKD and kidney failure combined were more than $138 billion in 2021, representing ∼29% of total Medicare expenditures for that year.^1^

The progression rate of CKD and the risk of CKD vary considerably among individuals. Most CKD remains underrecognized as a problem until the estimated glomerular filtration rate (eGFR) is <30-45 mL/min/1.73 m^2^.^2^ At these levels of kidney function, the window to initiate highly effective guideline medical therapy to slow progression is limited, and there are higher risks of side effects including hyperkalemia and acute kidney injury. As such, interventions that delay the progression of CKD need to be implemented early for maximum benefit and potential lifetime risk reduction for kidney failure. New methods for accurately identifying those at high risk of progression in earlier stages of CKD are needed.

The Kidney Failure Risk Equation (KFRE) is an internationally validated risk prediction equation that accurately predicts the risk of progression to kidney failure for an individual patient with CKD.^3^ However, the KFRE is only validated in individuals with stages G3-G5 CKD and predicts dialysis, which is a late outcome in CKD progression. To address this, we developed the Klinrisk machine learning model for prediction of the composite outcome of 40% decline in eGFR or kidney failure at all stages of CKD, including G1 and G2.^4^ The Klinrisk machine learning model uses routinely collected laboratory analytes and demographics and has been shown to accurately predict disease progression in large administrative datasets and global clinical trials.^4^, ^5^, ^6^, ^7^

Although previous work has shown that in later stages of disease, KFRE risk is associated with increased health care costs, less is known about the relationship between CKD progression risk in earlier stages of disease and costs of care. The purpose of the present analysis is to examine the association between risk of CKD progression as predicted by the Klinrisk model and costs of care at all stages of kidney disease.

## Methods

### Data Source

Data from Optum’s electronic health record (EHR) database covering the period of January 1, 2007 to September 30, 2022 were used.^8^ The database represents over 150,000 medical providers from more than 2,000 hospitals and 7,000 clinics and comprises medical records including information on demographic characteristics, medical histories and diagnoses, procedures, medications, and laboratory tests.

This study was considered exempt research under 45 Code of Federal Regulations §46.104(d)(4)^9^ as it involved only the secondary use of data that were deidentified in compliance with the Health Insurance Portability and Accountability Act, specifically, 45 Code of Federal Regulations §164.514. The research was conducted in accordance with the ethical principles of the Declaration of Helsinki.

### Study Design and Population

A retrospective observational cohort study design was used (Fig 1). Adult patients with CKD were identified either by evidence of impaired eGFR (ie, ≥2 eGFR measurements <60 mL/min/1.73 m^2^ taken 90-548 days apart) or abnormal urinary albumin-creatinine ratio (UACR; ≥1 UACR measurement ≥30 mg/g). The index date was defined as the first date when patients met the above case definition of CKD. The baseline period was defined as the 12 months prior to the index date. Patients were followed from the index date to the earliest of the end of continuous eligibility, death, or the end of data cut.

**Figure 1:**
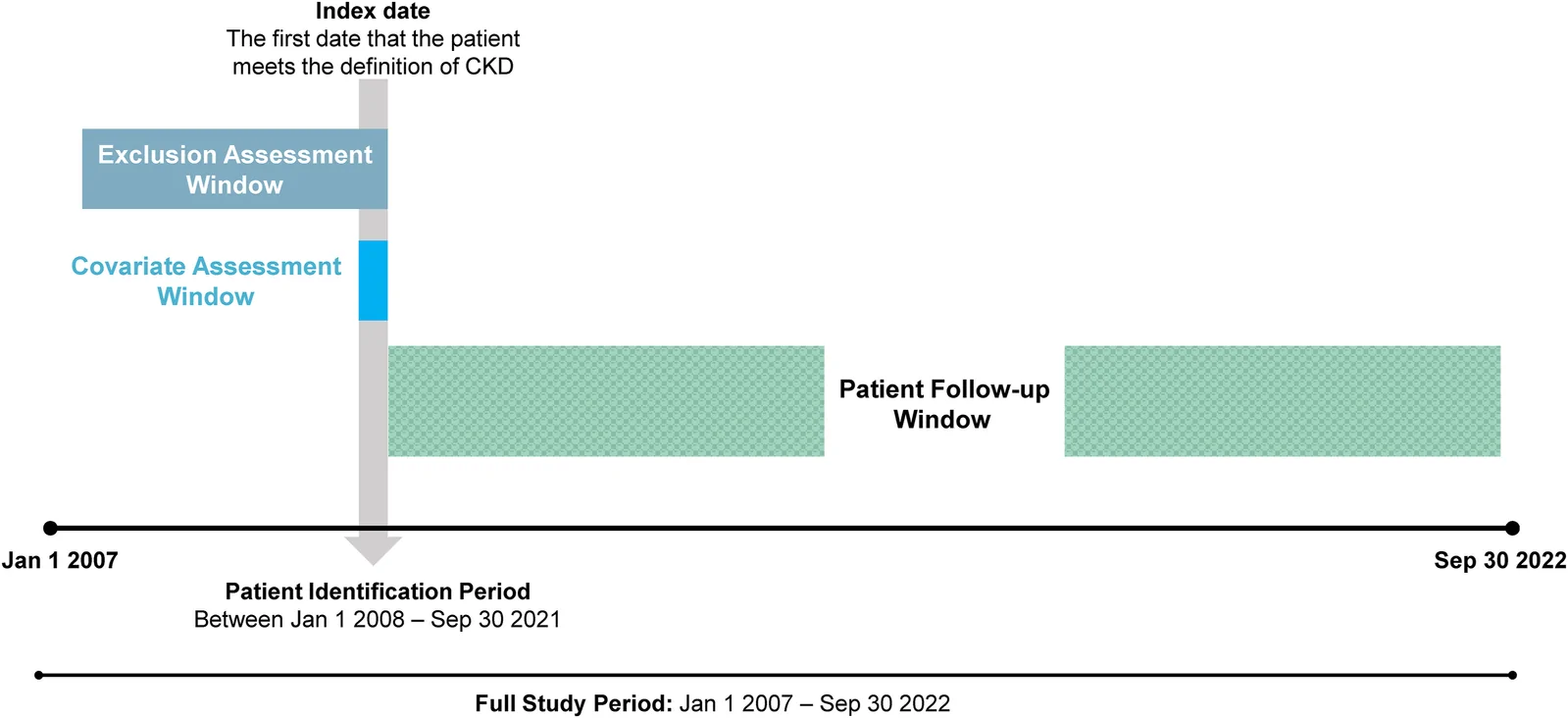
Study design schema. Abbreviation: CKD, chronic kidney disease.

Adult patients with CKD were included in the study if they met the following criteria: (1) had complete information on required predictors for the Klinrisk model (ie, age, sex, baseline eGFR, and baseline UACR^3^); (2) had ≥1 eGFR test after the index date; (3) had no history of kidney transplant or chronic dialysis prior to or on the index date (see Table S1 for diagnosis and procedure codes); and (4) had continuous activity in the data during the 12 months before and after the index date. Continuous activity was defined as <6-month gap between consecutive medical records or the last recorded activity date, whichever came first (to avoid selection bias, patients who died between 30 and 365 days post index were also included).

### Study Outcomes and Measures

The risk of CKD progression, defined as a composite outcome of 40% decline in eGFR or kidney failure,^4^ was predicted in the study population using the proprietary machine learning model Klinrisk. The Klinrisk model is a random forest survival model that was developed to predict CKD progression for patients at all stages of CKD. The model uses patient demographics and clinical characteristics as predictors, including age, sex, eGFR, UACR, and other routinely collected laboratory tests that are part of a chemistry panel, liver enzymes, and the complete blood panel.^3^ It has been validated with high accuracy in predicting CKD progression in both clinical trial data and general population.^5^ According to the Klinrisk-predicted risk of CKD progression, patients were categorized into 3 risk groups: low risk, defined as <2% risk at 2 years; medium risk, defined as 2%-10% risk at 2 years; and high risk, defined as >10% risk at 2 years.

For each Klinrisk-predicted risk group, baseline demographic and clinical characteristics, observed risk of CKD progression, and all-cause health care resource utilization (HRU) and medical costs were described. Specifically, all-cause HRU and medical costs were assessed during the 12 months post index or from the index date to the end of follow-up for those who died within 12 months. HRU and costs were reported annually per patient and by category as inpatient admissions, emergency room (ER) visits, and outpatient visits. As cost data were not directly available in the EHR, medical costs were estimated as the product of HRU frequencies and the corresponding unit costs (ie, inpatient: $4,424 per day; ER, $2,039 per visit; outpatient: $361 per visit) estimated using the Optum Clinformatics claims data from a previous study.^10^ Costs were adjusted for inflation using the medical component of Consumer Price Index for 2023 first-half US Dollars.^11^

### Statistical Analyses

The Klinrisk model predicted the risk of CKD progression based on patient demographics, clinical characteristics, and routine laboratory data in the EHR.^4^ Only age, sex, eGFR, and UACR were required variables. Data from other laboratory analyses, such as creatinine, urea, potassium, and values from complete blood cell count panels, could be included as optional predictors. The model also conducted imputations for missing data when these additional variables were not available.^12^ The performance of the Klinrisk model was assessed using the area under the receiver operating characteristic curve at 2 years. The model calibration was evaluated by plotting the Klinrisk-predicted risks against the observed risks of CKD progression by decile of the predicted risks.

To assess the risk categorization method using the Klinrisk model, patients were separately categorized into low-, medium-, and high-risk groups based on the Kidney Disease Improving Global Outcomes (KDIGO) heatmap risk categories at baseline.^13^ The agreement between the 2 risk categorization methods was compared using 2-way cross-tabulations and Cohen’s weighted κ with quadratic weights, where κ values can be interpreted as follows: ≤0 as indicating no agreement, 0.01-0.20 as none to slight, 0.21-0.40 as fair, 0.41-0.60 as moderate, 0.61-0.80 as substantial, and 0.81-1.00 as almost perfect agreement. In addition, among patients with ≥2 years of follow-up who experienced CKD progression within 2 years, a 2-way cross-tabulation was used to assess the reclassification performance of the Klinrisk model by comparing the KDIGO risk categories with the Klinrisk risk categories.

Descriptive statistics were used to summarize baseline patient characteristics and annual all-cause HRU and medical costs by Klinrisk-predicted risk group using means and standard deviations or medians and interquartile ranges for continuous variables and counts and percentages for categorical variables. The magnitude of the difference in baseline characteristics was assessed using standardized mean differences computed between the low- and medium-risk groups and between the medium- and high-risk groups.^14^

Kaplan–Meier analysis was used to describe the observed risk of CKD progression by Klinrisk-predicted risk group. The estimated probability of CKD progression in each risk group was reported at 1-14 years post index. Patients who did not experience CKD progression were censored at the end of the follow-up date.

All statistical analyses were performed using SAS version 9.4 (SAS Institute) and R 4.2.2 (R Core Team) software.

## Results

### Sample Size and Risk Group Classification

A total of 1,050,552 patients met the study criteria and were included in the analysis (Fig 2). Based on the Klinrisk model-predicted 2-year risk of CKD progression, 300,222 (28.6%) patients were classified as low risk, 618,600 (58.9%) as medium risk, and 131,730 (12.5%) as high risk.

**Figure 2:**
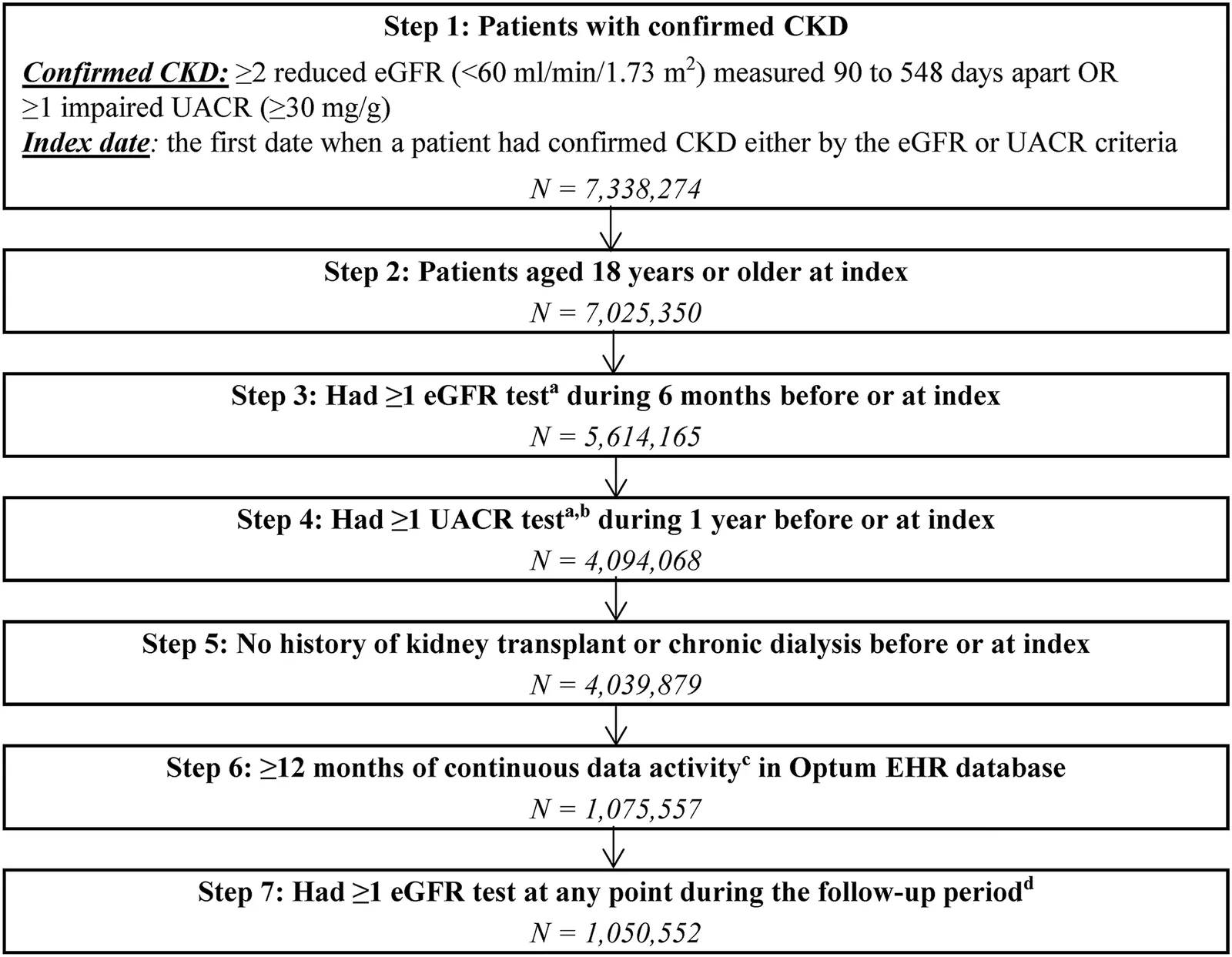
Sample selection flowchart. Abbreviations: CKD, chronic kidney disease; eGFR, estimated glomerular filtration rate; EHR, electronic health record; PCR, protein-creatinine ratio; UACR, urinary albumin-creatinine ratio; UPCR; urinary protein-creatinine ratio. ^a^Results from inpatient or emergency room settings are not included. ^b^If UACR test was unavailable, UACR was estimated using the UPCR test results, or if unavailable, urine dipstick test results using the following equations: for UPCR to UACR: exp(5.3920 + 0.3072 × log(min (PCR/50, 1)) + 1.5793 × log(max (min(PCR/500, 1), 0.1)) + 1.1266 × log(max (PCR/500, 1))); and for dipstick to UACR: exp(2.4738 + 0.7539 × (if trace) + 1.7243 × (if +) + 3.3475 × (if ++) + 4.6399 × (if >++)). ^c^A patient was considered continuously active in the data until either a 6-month gap between consecutive medical records or the last recorded activity date, whichever was earliest. ^d^Follow-up was defined as the time from the index date to the end of continuous enrollment, death, or end of data cutoff, whichever was earliest.

The Klinrisk model for predicting 2-year risk of CKD progression had an area under the receiver operating characteristic curve of 0.780 (95% confidence interval, 0.780-0.780). The calibration plot showed that in each risk decile, the risk of CKD progression predicted by the Klinrisk model was slightly higher than the observed risk (Fig S1). Comparison of risk categorizations using the Klinrisk model and the KDIGO heatmap showed agreement in 45.4% of patients, with a κ value of 0.34 (Table S2). Among patients who experienced CKD progression within 2 years, 39.7% of them were incorrectly classified as low risk according to the KDIGO risk categories but were more accurately reclassified into a higher risk group by the Klinrisk model. In contrast, only 2.7% of patients who experienced CKD progression within 2 years and correctly classified as high risk by KDIGO were reclassified into a lower-risk group by Klinrisk (Table S3).

### Baseline Patient Characteristics by Klinrisk-predicted Risk Group

Baseline patient characteristics by Klinrisk-predicted risk group are presented in Table 1. Patients at higher risk of CKD progression were generally older (mean [standard deviation] age in years: low risk: 52.0 [16.2]; medium risk: 62.9 [14.7]; high risk: 67.0 [13.3]). Across risk groups, there was a slightly higher proportion of female patients (54.6%-65.7%), and the most common race was Caucasian (80.3%-82.6%), followed by African American (11.2%-12.5%) and Asian (1.2%-2.1%); common comorbid conditions included hypertension (50.5%-63.9%) and type 2 diabetes (36.8%-40.9%).

**Table 1:** Baseline Patient Characteristics by Klinrisk-predicted Risk Group^a^

	Low risk N = 300,222 (A)	Medium risk N = 618,600 (B)	High risk N = 131,730 (C)	SMD (A) vs (B)	SMD (B) vs (C)
**Demographics**					
Age at index date, y	52.0 ± 16.2	62.9 ± 14.7	67.0 ± 13.3	0.701	0.295
Sex				0.136	0.091
Female	197,292 (65.7%)	365,736 (59.1%)	71,969 (54.6%)		
Male	102,930 (34.3%)	252,864 (40.9%)	59,761 (45.4%)		
Race				0.072	0.076
Caucasian	242,761 (80.9%)	511,203 (82.6%)	105,734 (80.3%)		
African American	33,742 (11.2%)	69,501 (11.2%)	16,440 (12.5%)		
Asian	6,258 (2.1%)	8,916 (1.4%)	1,618 (1.2%)		
Other/unknown	17,461 (5.8%)	28,980 (4.7%)	7,938 (6.0%)		
Region				0.050	0.046
Midwest	176,015 (58.6%)	374,064 (60.5%)	78,021 (59.2%)		
Northeast	46,248 (15.4%)	92,261 (14.9%)	21,715 (16.5%)		
South	46,367 (15.4%)	90,982 (14.7%)	19,015 (14.4%)		
West	20,481 (6.8%)	42,645 (6.9%)	9,311 (7.1%)		
Other/unknown	11,111 (3.7%)	18,648 (3.0%)	3,668 (2.8%)		
Health insurance provider					
Commercial	222,515 (74.1%)	397,880 (64.3%)	73,843 (56.1%)	0.213	0.169
Medicare	78,364 (26.1%)	285,201 (46.1%)	71,412 (54.2%)	0.426	0.163
Medicaid	55,615 (18.5%)	87,734 (14.2%)	20,813 (15.8%)	0.118	0.045
Uninsured	47,704 (15.9%)	81,815 (13.2%)	17,548 (13.3%)	0.076	0.003
Other payer type	15,558 (5.2%)	30,607 (4.9%)	7,497 (5.7%)	0.011	0.033
Unknown	34,938 (11.6%)	76,145 (12.3%)	18,412 (14.0%)	0.021	0.049
**Comorbid conditions**					
Hypertension	151,695 (50.5%)	381,015 (61.6%)	84,230 (63.9%)	0.224	0.049
Type 2 diabetes	110,399 (36.8%)	253,188 (40.9%)	49,590 (37.6%)	0.085	0.067
Heart failure	6,465 (2.2%)	36,262 (5.9%)	19,318 (14.7%)	0.190	0.293
Stroke	8,369 (2.8%)	31,950 (5.2%)	10,182 (7.7%)	0.122	0.105
Myocardial infarction	6,325 (2.1%)	24,699 (4.0%)	9,940 (7.5%)	0.110	0.153
Type 1 diabetes	7,995 (2.7%)	16,327 (2.6%)	4,583 (3.5%)	0.001	0.049
**Medication use**					
ACEi	67,169 (22.4%)	168,228 (27.2%)	35,455 (26.9%)	0.112	0.006
ARB	36,967 (12.3%)	90,374 (14.6%)	17,570 (13.3%)	0.067	0.037
SGLT2i	6,019 (2.0%)	10,424 (1.7%)	987 (0.7%)	0.024	0.085
Steroidal MRA	5,197 (1.7%)	16,248 (2.6%)	6,594 (5.0%)	0.061	0.124
GLP-1 RA	8,703 (2.9%)	15,431 (2.5%)	1,958 (1.5%)	0.025	0.072
**Laboratory test results**^b^**^,^**^c^					
eGFR, mL/min/1.73 m^2^	94.5 ± 21.7	80.7 ± 21.1	63.7 ± 25.5	0.642	0.729
eGFR stages				0.562	0.734
G1 (≥90 mL/min/1.73 m^2^)	183,536 (61.1%)	219,639 (35.5%)	21,739 (16.5%)		
G2 (60-89 mL/min/1.73 m^2^)	85,472 (28.5%)	276,588 (44.7%)	51,538 (39.1%)		
G3a (45-59 mL/min/1.73 m^2^)	29,675 (9.9%)	95,538 (15.4%)	24,989 (19.0%)		
G3b (30-44 mL/min/1.73 m^2^)	1,536 (0.5%)	25,203 (4.1%)	18,719 (14.2%)		
G4 (15-29 mL/min/1.73 m^2^)	3 (0.0%)	1,632 (0.3%)	12,290 (9.3%)		
G5 (<15 mL/min/1.73 m^2^)	0 (0.0%)	0 (0.0%)	2,455 (1.9%)		
UACR, mg/g, median (IQR)	66.6 (37.2, 66.6)	66.6 (45.5, 122.0)	337.4 (66.6, 877.1)	0.423	0.604
UACR stages				0.440	0.813
A1 (<30 mg/g)	27,493 (9.2%)	69,642 (11.3%)	11,209 (8.5%)		
A2 (30-300 mg/g)	256,821 (85.5%)	433,438 (70.1%)	48,528 (36.8%)		
A3 (>300 mg/g)	15,908 (5.3%)	115,520 (18.7%)	71,993 (54.7%)		
Serum potassium, mmol/L	4.1 ± 0.4	4.2 ± 0.5	4.3 ± 0.6	0.140	0.114
Blood urea nitrogen, mmol/L	4.9 ± 1.6	6.1 ± 2.4	9.6 ± 5.5	0.626	0.813
Serum albumin, g/L	41.4 ± 4.4	40.2 ± 4.6	35.6 ± 6.5	0.265	0.805
Serum calcium, mmol/L	2.3 ± 0.1	2.3 ± 0.1	2.3 ± 0.2	0.065	0.427
Hemoglobin, g/L	139.2 ± 13.5	133.8 ± 16.7	114.5 ± 20.4	0.355	1.035
Blood glucose, mmol/L	6.7 ± 2.8	7.3 ± 3.5	7.7 ± 4.2	0.187	0.110
Serum bicarbonate, mmol/L	25.6 ± 2.9	26.0 ± 3.4	25.0 ± 4.9	0.114	0.239
Missing	284,568 (94.8%)	579,420 (93.7%)	118,574 (90.0%)		
Aspartate aminotransferase, IU/L	24.6 ± 15.3	26.9 ± 27.9	35.4 ± 55.5	0.106	0.192
Alanine aminotransferase, IU/L	28.7 ± 27.2	29.2 ± 50.9	36.4 ± 119.9	0.013	0.077
Alkaline phosphatase, IU/L	79.8 ± 29.0	84.4 ± 41.6	105.4 ± 90.9	0.128	0.297
Serum phosphate, mmol/L	1.1 ± 0.2	1.1 ± 0.2	1.2 ± 0.3	0.056	0.344
Missing	283,134 (94.3%)	566,936 (91.6%)	100,860 (76.6%)		
Serum chloride, mmol/L	102.7 ± 3.0	102.3 ± 3.9	102.6 ± 5.5	0.126	0.064

*Note:* Data are presented as mean ± standard deviation for continuous variables or count (percentage) for categorical variables, unless otherwise mentioned.

Abbreviations: ACEi, angiotensin-converting enzyme inhibitor; ARB, angiotensin II receptor blocker; eGFR, estimated glomerular filtration rate; GLP-1 RA, glucagon-like peptide-1 receptor agonist; IQR, interquartile range; MRA, mineralocorticoid receptor antagonist; SMD, standardized mean difference; SGLT2i, sodium/glucose cotransporter 2 inhibitor; UACR, urinary albumin-creatinine ratio.

a Risk groups were classified based on the 2-year risk of chronic kidney disease progression predicted by the Klinrisk model: low risk, <2% risk at 2 years; medium risk, 2%-10% risk at 2 years; and high risk, >10% risk at 2 years.

b Results from inpatient or emergency room settings are not included. Missing rates are reported when data are missing in >30% of the patients.

c eGFR was computed as the average of all eGFR results during the 6 months before and including the index date. For other laboratory results, the closest results during the 12 months before and including the index date were used.

During the baseline period, patients in higher risk groups had lower mean eGFR (low risk: 94.5; medium risk: 80.7; high risk: 63.7 mL/min/1.73 m^2^) and considerably higher median UACR (low risk: 66.6; medium risk: 66.6; high risk: 337.4 mg/g). In all risk groups, the use of disease-controlling medications at baseline was generally low. Among low-, medium-, and high-risk patients, 22.4%, 27.2%, and 26.9%, respectively, used angiotensin-converting enzyme inhibitors; 12.3%, 14.6%, and 13.3%, respectively, used angiotensin II receptor blockers; and across risk groups, ≤5.0% used steroidal mineralocorticoid receptor antagonists, sodium/glucose cotransporter 2 inhibitors, and glucagon-like peptide-1 receptor agonists.

### Observed Risk of CKD Progression by Klinrisk-predicted Risk Group

Overall, the observed risk of CKD progression over time aligned with the risk profiles indicated by the Klinrisk risk category; from 1 to 14 years post index, the estimated probability of CKD progression was the lowest in the low-risk group and highest in the high-risk group (Fig 3).

**Figure 3:**
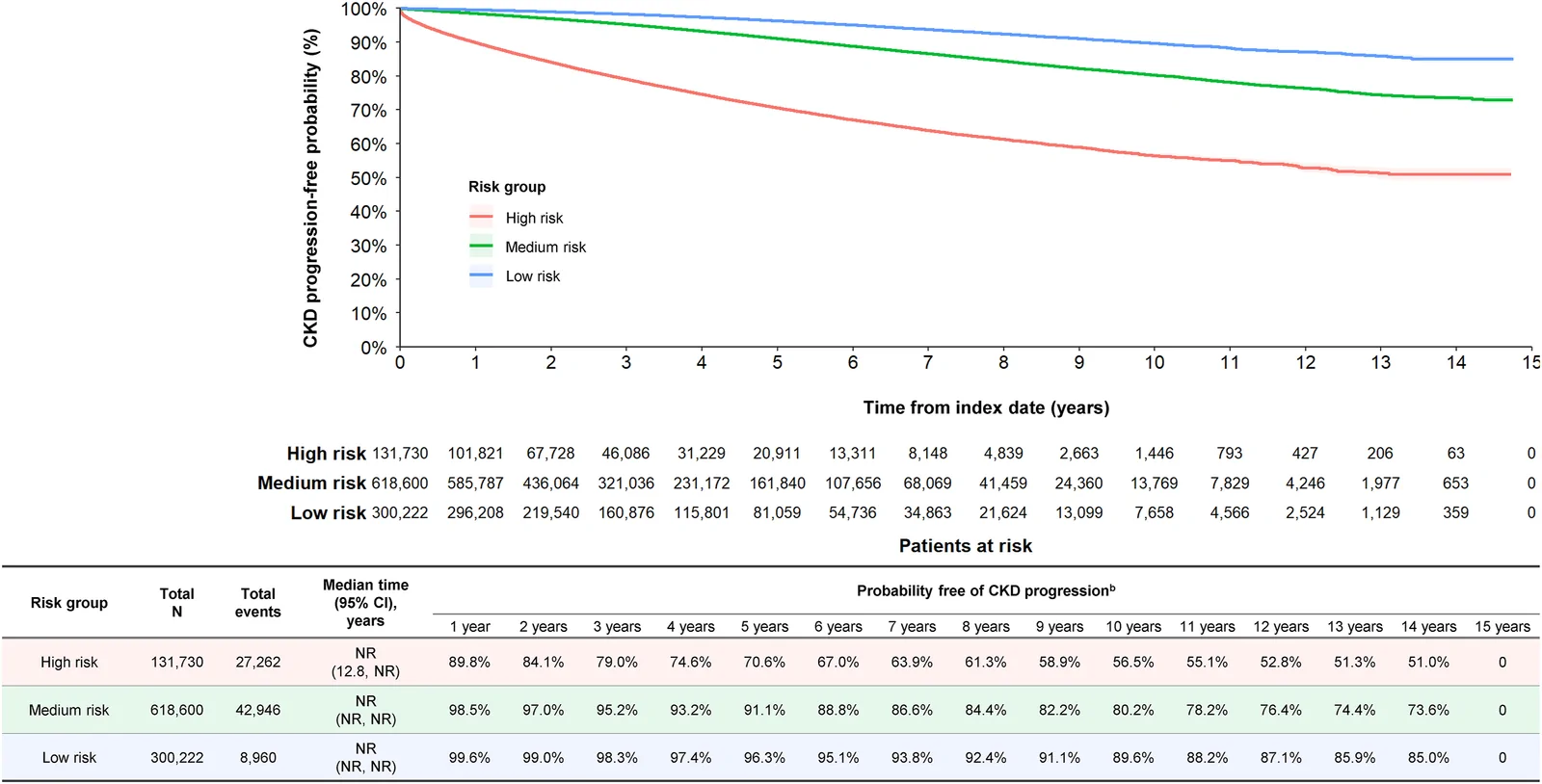
Kaplan–Meier analysis of time to CKD progression by risk group. Abbreviations: CI, confidence interval; CKD, chronic kidney disease; eGFR, estimated glomerular filtration rate; NR, not reached. ^a^Risk groups were classified based on the 2-year risk of CKD progression predicted by the Klinrisk model: low risk, <2% risk at 2 years; medium risk, 2%-10% risk at 2 years; and high risk, >10% risk at 2 years. ^b^CKD progression was defined as the onset of kidney failure (ie, the initiation of chronic dialysis, receipt of a transplant, or an eGFR <10 mL/min/1.73 m^2^), or 40% decline in eGFR (ie, the first eGFR test in the laboratory data that was ≥40% in decline from the baseline eGFR, with a second confirmatory test result between 90 days and 2 years after the first test unless the patient died or experienced kidney failure within 90 days after the first test).

At 2 years, the observed rate of CKD progression was 15.9% in the high-risk group, 4.8% in the medium-risk group, and 1.0% in the low-risk group. The observed rate of CKD progression at 5 and 10 years increased to 29.4% and 43.5% in the high-risk group, 8.9% and 19.8% in the medium-risk group, and 3.7% and 10.4% in the low-risk group, respectively.

### All-Cause HRU and Medical Costs by Klinrisk-predicted Risk Group

All-cause HRU and medical costs were generally higher among patients at a higher risk of CKD progression. Specifically, patients in the high-risk group had an average of 1.49 inpatient admissions, 0.83 ER visits, and 35.75 outpatient visits annually, whereas these HRU incidences averaged 0.51, 0.58, and 27.31 for those in the medium-risk group and 0.31, 0.63, and 24.45 for those in the low-risk group annually (Table 2).

**Table 2:** Annual All-Cause HRU in Patients With CKD Stratified by Klinrisk-predicted Risk Group^a^

	Low risk N = 300,222	Medium risk N = 618,600	High risk N = 131,730
HRU assessment duration^b^, y	1.00 ± 0.05	0.98 ± 0.11	0.91 ± 0.23
**Inpatient admissions**			
Patients with ≥1 inpatient admission	56,808 (18.92%)	133,921 (21.65%)	50,545 (38.37%)
Number of inpatient admissions	0.31 ± 1.00	0.51 ± 1.79	1.49 ± 3.90
Hospitalization duration, d	1.34 ± 6.23	2.50 ± 10.98	8.06 ± 23.74
**ER visits**			
Patients with ≥1 ER visit	73,778 (24.57%)	148,798 (24.05%)	38,915 (29.54%)
Number of ER visits	0.63 ± 2.08	0.58 ± 1.95	0.83 ± 2.33
**Outpatient visits**			
Patients with ≥1 outpatient visit	299,079 (99.62%)	615,645 (99.52%)	130,520 (99.08%)
Number of outpatient visits	24.45 ± 20.25	27.31 ± 23.41	35.75 ± 32.48

*Note:* Data are presented as mean ± standard deviation for continuous variables or count (percentage) for categorical variables.

Abbreviations: CKD, chronic kidney disease; ER, emergency room; HRU, health care resource utilization.

a Risk groups were classified based on the 2-year risk of CKD progression predicted by the Klinrisk model: low risk, <2% risk at 2 years; medium risk, 2%-10% risk at 2 years; and high risk, >10% risk at 2 years.

b HRU was assessed during the 12 months post index or from the index date to the end of follow-up for those who died within 12 months.

Annual all-cause medical costs in the high-risk group were $50,218 per patient, more than double the costs in the medium-risk group ($22,090) and triple those in the low-risk group ($16,018) (Fig 4^10^). For patients in the high-risk group, the main cost driver was inpatient costs ($35,643, 71.0% of total costs), followed by outpatient costs ($12,891, 25.7%) and ER costs ($1,684, 3.4%). Meanwhile, inpatient and outpatient costs were more comparable for patients in the medium-risk (inpatient: $11,059, 50.0%; outpatient: $9,846, 44.6%) and low-risk (inpatient: $5,915, 36.9%; outpatient: $8,815, 55.0%) groups.

**Figure 4:**
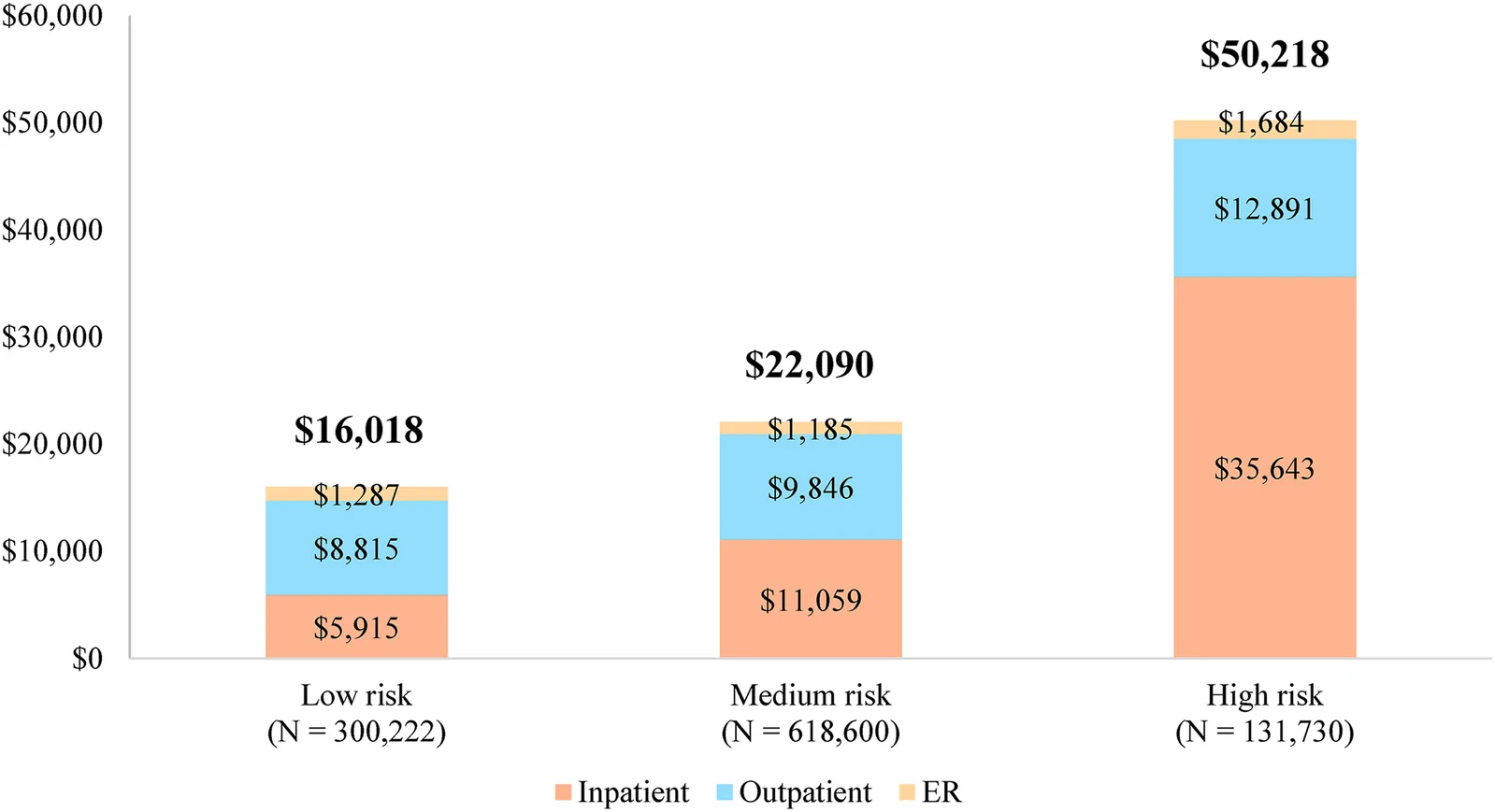
Annual all-cause medical costs in patients with CKD by risk group. Abbreviations: CKD, chronic kidney disease; ER, emergency room; HRU, health care resource utilization. ^a^Risk groups were classified based on the 2-year risk of CKD progression predicted by the Klinrisk model: low risk, <2% risk at 2 years; medium risk, 2%-10% risk at 2 years; and high risk, >10% risk at 2 years. ^b^Average medical costs were calculated as the product of health care resource utilization frequencies and the corresponding unit costs (ie, inpatient: $4,424 per day; ER, $2,039 per visit; outpatient: $361 per visit) estimated using the Optum Clinformatics claims data from a previous study.^10^

## Discussion

In this study of more than 1 million US patients with CKD, we found that higher risk of CKD progression as determined by the Klinrisk model was associated with increased annual all-cause medical costs per patient. Furthermore, the Klinrisk model demonstrated good discriminative ability (area under the curve, 0.78) and improved risk stratification over the KDIGO heatmap by correctly reclassifying nearly 40% of patients who experienced CKD progression within 2 years but were initially classified as low risk using the KDIGO heatmap. Taken together, these findings suggest that the Klinrisk model can be used to identify patients at the highest risk of progression, and the highest costs of care in a US health-insured population. These patients represent the ideal candidates for intervention, which would potentially reduce costs and improve health outcomes.

The Klinrisk model uses demographic and laboratory inputs that are routinely collected and widely available in EHR or laboratory information systems. Research has shown that the cost of care per patient increases as disease progresses, being the highest in those reaching kidney failure.^15^ Our previous work using the KFRE showed that in patients with later stages of CKD (G3-G4), further risk stratification with the KFRE resulted in discrimination between overall costs of care within each CKD stage.^16^ This study greatly expands upon our previous work by adding patients with earlier stages of disease and by studying costs in nearly 1 million US individuals, compared with our previous work in 1,000 Canadians. In addition, previous research showed that the Klinrisk model was able to accurately identify patients with type 2 diabetes and CKD who required the most health care resources,^17^^,^^18^ and the current study further demonstrates the utility of the Klinrisk model in the general CKD population. It is important to note that despite lower eGFRs and higher albuminuria values in high-risk individuals, more than half of the identified high-risk population in this study had an eGFR >60 mL/min/1.73 m^2^, and 45% had microalbuminuria or absence of albuminuria. In current clinical practice, these patients are largely considered low risk, often receive no formal diagnosis, minimal intervention, and only usual follow-up for their unrecognized CKD. Our work and work by others on the Chronic Kidney Disease Prognosis Consortium model shows that approximately 4.77 million Americans are high risk for CKD progression despite having normal eGFR values. Risk prediction models remain the only method to identify these individuals early, allowing for maximal benefit.^19^

Early intervention with disease-modifying therapies such as sodium/glucose cotransporter 2 inhibitors, nonsteroidal mineralocorticoid receptor antagonists, and glucagon-like peptide-1 receptor agonists has been shown to effectively slow progression of kidney disease and prevent cardiovascular events.^20^, ^21^, ^22^, ^23^ In addition, value-based care organizations have deployed additional resources including nurse care managers, digital tools, and home visits to prevent progression and reduce hospitalizations and overall costs. These efforts continue to be largely focused only on patients with severely reduced eGFR (<30 mL/min/1.73 m^2^), with very poor recognition of high-risk disease in patients with preserved kidney function.

In the United States, these disease-modifying therapies are expensive, and so are the human resources required for care management models. As such, health systems and value-based care organizations need to triage and prioritize the delivery of resources to patients with highest risk and cost. As an example, patients identified as high or intermediate risk using the Klinrisk tool could receive 3-4 drugs to slow progression and receive in-person case management such as a home visit, whereas those at lower risk could continue to receive renin-angiotensin-aldosterone system and sodium/glucose cotransporter 2 inhibitor therapies only and be provided CKD education using a lower cost digital offering. Given that approximately half of all health spending in the United States is incurred by 5% of patients,^24^ our data would suggest that some triage of the highest priority members is needed to reduce costs.

Our study has several strengths. It included 1,050,552 adults across the United States within the Optum EHR database, supporting its generalizability to the broader CKD population. Moreover, we demonstrated the improved risk classification with the Klinrisk model compared with the current standard of care (KDIGO heatmap). This study now extends the validation of the model to 5 million individuals worldwide ranging from clinical trial populations of people with diabetic and nondiabetic CKD to a lower risk commercial insurance population that is largely CKD-free.^7^ Our study also has limitations. First, our analysis only considered direct medical costs to the payer and omitted indirect costs (eg, transportation, capital, health records). Second, medical costs were not available in the EHR and thus estimated costs were used, which may not reflect actual costs paid. Relatedly, the cost estimates reported herein may not directly translate to other non-US health care systems due to variations in health care delivery models, reimbursement structures, and unit costs; nonetheless, the observed risk of CKD progression and gradient of increasing health care burden with higher CKD progression risk would be expected to persist. Third, the model predicts CKD progression and does not overtly predict cardiovascular disease outcomes. However, work from others has shown that patients identified as high risk for CKD have the lowest number needed to treat for combined cardiorenal outcomes compared with low or intermediate risk populations. In an analysis from the CANVAS/CREDENCE trials, high-risk (Klinrisk) patients had a number needed to treat of 4 for the primary outcome (cardiovascular + renal) compared with >100 for lower-risk individuals.^6^ Finally, we demonstrated improvements in albuminuria testing and guideline-directed medical therapy use with implementation of the Klinrisk model,^25^ but data on reduced health care costs with model implementation are still pending and should be studied.

In conclusion, we demonstrate a strong association with risk of CKD progression, as calculated with the Klinrisk model, and overall costs of care. Patients identified as high risk progress rapidly and incur a disproportionate burden of hospitalization. Prospective implementation studies of the model and its effect on determining care pathways and downstream cost reduction in CKD management are needed.
