# Supplementary material for: Risk of Progression and Costs of Care for Patients With Chronic Kidney Disease
Source: Kidney Med. 2026 Aug 3;8(10):101484. doi: 10.1016/j.xkme.2026.101484 (PMC13625882; doi:10.1016/j.xkme.2026.101484)
Supplement: Supplementary file 1 [file mmc1.pdf]

SUPPLEMENTARY MATERIAL

Table S1. Diagnosis and procedure codes for kidney transplant and chronic dialysis

Procedure	Coding classification system	Codes
Kidney transplant	ICD-9-CM, Vol 1 or 2 (diagnosis codes)	996.81, V42.0
	ICD-10-CM	T86.10, T86.11, T86.12, T86.13, T86.19, Z94.0, Z48.22
	ICD-9-CM, Vol 3 (procedure codes)	00.91, 00.92, 00.93, 55.53, 55.61, 55.69
	ICD-10-PCS	0TY00Z0, 0TY00Z1, 0TY00Z2, 0TY10Z0, 0TY10Z1, 0TY10Z2
	DRG	652, 008
	CPT4	50360, 50365, 50370, 50380
Chronic dialysis	ICD-9-CM, Vol. 1 or 2 (diagnosis codes)	585.6, V45.11, V56.0, V56.1, V56.2, V56.31, V56.32, V56.8
	ICD-10-CM codes	N18.6, Z99.2, Z49.31, Z49.01, Z49.02, Z49.31, Z49.32, Y84.1
	CPT4	90918, 90919, 90920, 90921, 90922, 90923, 90924, 90925, 90935, 90937, 90940, 90945, 90947, 90951, 90952, 90953, 90954, 90955, 90956, 90957, 90958, 90959, 90960, 90961, 90962, 90963, 90964, 90965, 90966, 90967, 90968, 90969, 90970, 90989, 90993, 90997, 90999
	HCPCS	G0257, G8081, G8082, G8085, G8714, G8715, G8956, G9264, G9265, G9266, S9335, S9339, G0491, G0492<br><br>*Codes used in claims before 1/1/2009:*<br><br>G0317, G0318, G0319, G0320, G0321, G0322, G0323, G0324, G0325, G0326, G0327
	ICD-9-CM, Vol 3 (procedure codes)	38.95, 39.27, 39.42, 39.95, 54.98
	ICD-10-PCS	5A1D00Z, 5A1D60Z, 3E1M39Z, 5A1D70Z, 5A1D80Z, 5A1D90Z

Abbreviations: CPT, Current Procedural Terminology; DRG, Diagnosis-Related Group; HCPCS, Healthcare Common Procedure Coding System; ICD-9/10-CM, International Classification of Diseases, 9th/10th Revision, Clinical Modification; ICD-10-PCS, International Classification of Diseases, 10th Revision, Procedure Coding System.

Table S2. Number and percentage of patients assigned to each CKD progression risk group by Klinrisk risk and KDIGO risk[a-c]

Kappa = 0.34		KDIGO risk		
		**Low**	**Medium**	**High**
**Klinrisk, 2-year**	**Low**	279,249 (26.6%)	20,777 (2.0%)	196 (0.0%)
	**Medium**	443,501 (42.2%)	155,540 (14.8%)	19,559 (1.9%)
	**High**	34,303 (3.3%)	55,717 (5.3%)	41,710 (4.0%)

Abbreviations: CKD: chronic kidney disease; KDIGO: Kidney Disease Improving Global Outcomes.

[a] The Klinrisk risk groups were based on the 2-year risk of CKD progression predicted by the Klinrisk model and were defined as follows: low risk: <2% at 2 years; medium risk: 2%–10% at 2 years; high risk: >10% at 2 years.

[b] The KDIGO risk groups were defined according to the KDIGO heatmap, evaluated at baseline, with yellow entries corresponding to low risk, orange entries corresponding to medium risk, and red entries corresponding to high risk.

[c] Cohen's weighted kappa with quadratic weights was used as a measure of agreement between different risk criteria. Compared to the unweighted kappa, the weighted kappa penalizes disagreements in terms of their seriousness and is more suitable for ordinal categories. The kappa statistics can be interpreted as follows: kappa ≤ 0 as indicating no agreement, 0.01–0.20 as none to slight, 0.21–0.40 as fair, 0.41– 0.60 as moderate, 0.61–0.80 as substantial, and 0.81–1.00 as almost perfect agreement.

Table S3. Reclassification table comparing KDIGO risk groups to Klinrisk risk groups of CKD progression, among patients who experienced CKD progression within 2 years[a-d]

		Klinrisk, 2-year			Percentage of patients reclassified into a new risk category (KDIGO risk to Klinrisk risk)	
		**Low**	**Medium**	**High**	**Lower**	**Higher**
**KDIGO risk**	**Low**	2,064 (8.3%)	8,020 (32.4%)	1,815 (7.3%)	-	39.7%
	**Medium**	118 (0.5%)	3,025 (12.2%)	3,287 (13.3%)	0.5%	13.3%
	**High**	4 (0.0%)	661 (2.7%)	5,791 (23.4%)	2.7%	-

Abbreviations: CKD: chronic kidney disease; KDIGO: Kidney Disease Improving Global Outcomes.

[a] The Klinrisk risk groups were based on the 2-year risk of CKD progression predicted by the Klinrisk model: low risk, <2% risk at 2 years; medium risk, 2%–10% risk at 2 years; and high risk, >10% risk at 2 years.

[b] The KDIGO risk groups were defined according to the KDIGO heatmap, evaluated at baseline, with yellow entries corresponding to low risk, orange entries corresponding to medium risk, and red entries corresponding to high risk.

[c] Patients included experienced CKD progression within 2 years and had at least 2 years of follow-up (N=24,785).

[d] Percentages of reclassification were calculated with respect to the total number of overall patients, cases, and controls, respectively.

Figure S1. Calibration of the Klinrisk model for prediction of risk of CKD progression at 2 years[a]

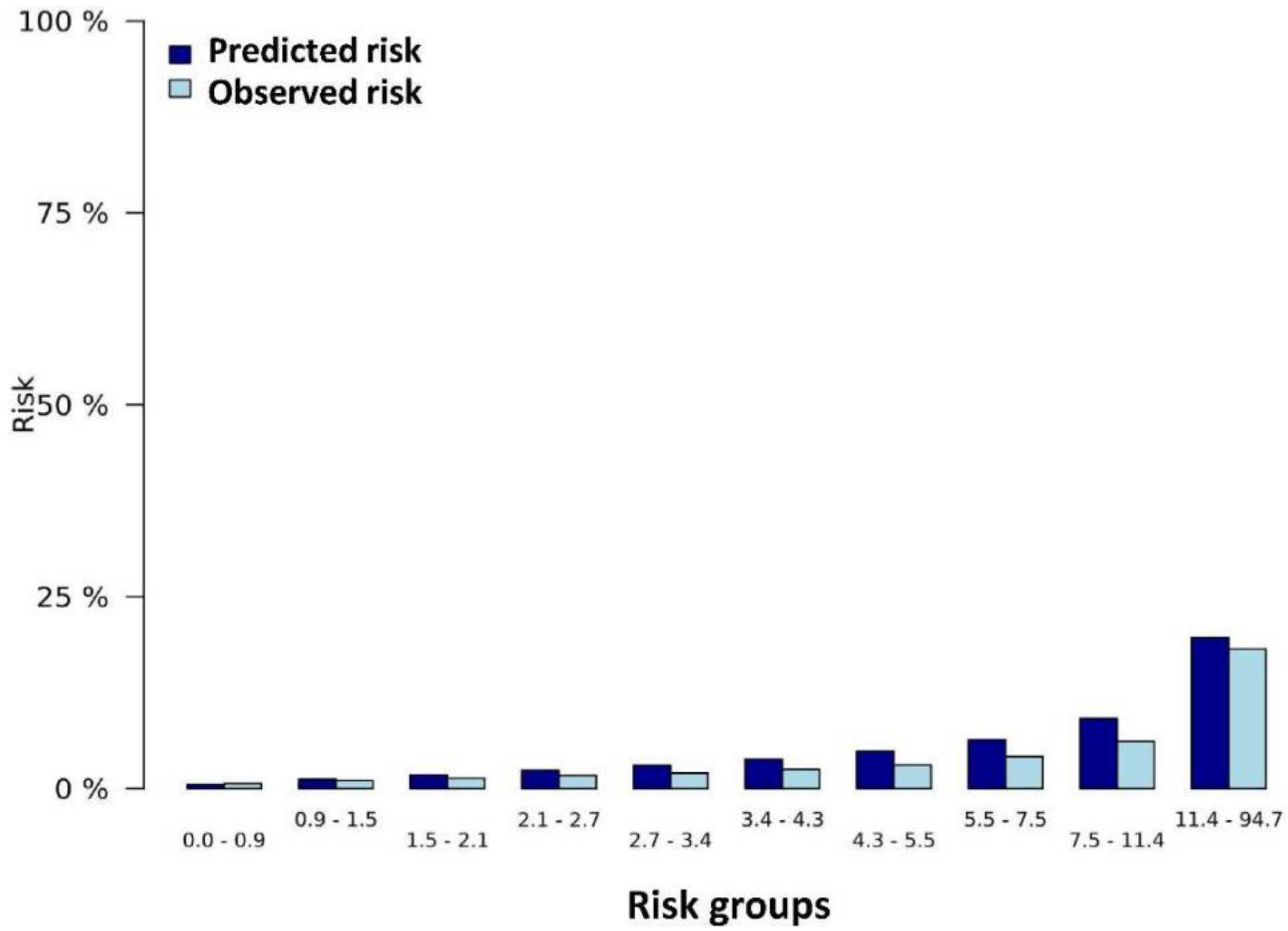

Abbreviations: CKD: chronic kidney disease; eGFR, estimated glomerular filtration rate.

[a] The observed risk of CKD progression based on Kaplan–Meier analysis, wherein the CKD progression was defined as the onset of kidney failure (i.e., the initiation of chronic dialysis, receipt of a transplant, or an eGFR <10 ml/min/1.73m^2), or 40% decline in eGFR (i.e., the first eGFR test in the laboratory data that was 40% or greater in decline from the baseline eGFR, with a second confirmatory test result between 90 days and 2 years after the first test unless the patient died or experienced kidney failure within 90 days after the first test).
